# Functional Electrical Stimulation Alters the Postural Component of Locomotor Activity in Healthy Humans

**DOI:** 10.3389/fnins.2015.00478

**Published:** 2015-12-18

**Authors:** Vera Talis, Yves Ballay, Alexander Grishin, Thierry Pozzo

**Affiliations:** ^1^Institute for Information Transmission ProblemsMoscow, Russia; ^2^Institut National de la Santé et de la Recherche Médicale, U1093, Cognition Action Plasticité SensorimotriceDijon, France; ^3^Department of Robotics, Brain and Cognitive Sciences, Istituto Italiano di TecnologiaGenova, Italy; ^4^Université de Bourgogne, UFR STAPS (Sciences du Sport)Dijon, France

**Keywords:** FES, postural stability, locomotion, healthy subjects

## Abstract

Knowledge of the effects of Functional Electrical Stimulation (FES) of different intensity on postural stability during walking in healthy subjects is necessary before these relationships in patients with postural disorders can be assessed and understood. We examined healthy subjects in Control group walking on a treadmill for 40 min and in FES group—provided with 30 min of stimulation, which intensity increased every 10 min. The main difference between Control and FES group was the progressive increase of trunk oscillations in sagittal, frontal, and horizontal planes and an increase of relative stance duration in parallel with FES intensity increase. Both Control and FES groups exhibited shank elevation angle increase as an after-effect. It is concluded, that high intensity FES significantly changes the postural component of locomotor activity, but the fatigue signs afterwards were not FES specific.

## Introduction

Human walking is characterized by the repetitive motion of limb segments aimed to propel the body forward. Joint movement is highly synchronized and muscle activity displays a typical periodical pattern (Bernstein, 1990; Perry, 1992). These rhythmic locomotor movements are produced by a brainstem-spinal central pattern generator (CPG) that is activated by descending command signals (Grillner, 1981; Grasso et al., 1998; Selionov et al., 2009). Any unpredicted disturbance during walking, such as stumbling (Eng et al., 1994; Schilling et al., 2000), could be compensated through sensory feedback. In the experimental environment, the kinematics of walking have been shown to be well preserved even under the conditions of body weight unloading (Ivanenko et al., 2002), additional loading of the legs (Smith and Martin, 2007), and during “split-belt” locomotion (Jensen et al., 1998). As an example, Ivanenko et al. (2002) has shown that dynamical change, such as an artificial decrease of foot pressure, resulted in weak changes in the coordination of segments.

A specific type of gait disturbance is the direct stimulation of muscles at the time of muscle action during the step cycle. Such a periodical stimulation could be implemented by means of electrical or vibratory intervention. Ivanenko et al. (2000) analyzed the effects of phasic leg muscle vibration on human locomotion, and have shown that the vibration of hamstring muscles produced an increase of walking speed, while the vibration of other leg muscles did not. Based on the well-known fact of position and movement illusions caused by muscle vibration in an upright standing position (Lackner and Levine, 1979; Talis and Solopova, 2000), Ivanenko and colleagues explained that hamstring vibration induced a rise in speed through the modification of kinesthetic information about joint angels consistent with the lengthening of the hamstring muscle.

In this paper, we studied the effects of electrical phasic stimulation of leg muscles (Functional Electrical Stimulation, FES) during walking. In contrast to vibratory stimulation, electrical stimulation, directly affecting the relation between motor command and force output, ensures an almost immediate muscle contraction. This increases the precision of the application of the stimulus and thus allows the stimulation of several muscles in an alternative manner through each gait cycle. The force of the muscle response to the electrical stimulation can be increased to up to 70% of maximum voluntary contraction MVC (DeVahl, 1992) [vibration elicited not more than 30% of MVC of the stimulated muscle (Matthews, 1966)]. Walking speed increase with regular use of FES in spinal cord injury (Ladouceur and Barbeau, 2000; Pomeroy et al., 2006) and stroke patients (Lindquist et al., 2007) was reported. In was shown, that this gait speed advantage lasted from weeks to a month in stroke (Bogataj et al., 1995; Alon and Ring, 2003; Daly et al., 2011) and spinal cord injury patients (Ladouceur and Barbeau, 2000; for reviews see Barbeau et al., 2002). At the same time, the origin of the retention of an increased walking speed without continued FES, meaning the long lasting effect of FES-training, called the therapeutic effect of FES-assisted walking, is unclear and even discounted (see for instance the discussion about implantable electrode technology, as a possible future of electrical stimulation in Burridge and Hughes, 2010). For instance, post-stroke gait speed increase due to FES might be because walking rate reduction was the primary reason for FES use (Taylor et al., 1999).

The rationale of the present study was to study the kinematics of healthy subjects during treadmill locomotion and compare the results of two groups, one of which was simultaneously provided with FES (FES group), and the other was not (Control group). A short account of some of the present findings was published as an abstract (Talis et al., 2011). The collected data of FES-assisted walking in healthy subjects could be implemented in the pathology of FES-assisted walking.

## Materials and methods

### Subjects

Eight healthy subjects participated in the FES group [seven males and one female, between 25 and 49 year of age, 74 ± 11 (SD) kg, 1.76 ± 0.1 m] and eight subjects in the Control group (six males and two females, between 20 and 49 year of age, 73 ± 11 kg, 1.74 ± 0.09 m, five of them from the FES group). None of the subjects had any history of neurological disease or vestibular impairment. The experiments conformed to the Declaration of Helsinki and written informed consent was obtained from all the participants according to the protocol of the Ethics Committee of the Universite de Bourgogne.

### Experimental setup and stimulation techniques

The subjects walked on a treadmill at individually adjusted speed of about 0.7 m/s with their shoes on. Subjects from the FES group have eight bipolar stimulation surface electrodes (5 × 5 and 5 × 10 cm) placed bilaterally on four muscles [Tibialis Anterior (TA), Gastrocnemius Medialis (GM), Quadriceps (Q), and Biceps Femoris (BF) of both legs] with the negative electrode over a motor point (DeVahl, 1992). Electrical stimulus consisted of repetitive trains of rectangular pulses with 65 mA amplitude at 65 Hz. A custom-made eight-channel stimulator delivered the desired stimulation train, triggered by the signal of the right knee goniometer in such a way, that the timing of the stimulation sequence corresponded to the timing of the activation sequence of these muscles during normal gait (Perry, 1992).

### Data recording

Body kinematics was recorded by means of the ELITE system (BTS, Italy). Nine 120-Hz TV cameras were spaced around a treadmill in a 4 × 4 × 2 m acquisition volume. Hemispherical reflective markers of 15-mm diameter attached to the skin overlying the following body landmarks for the two hemibodies: laterally on the fifth metatarsophalangeal joint (MTP), lateral malleolus (MAL), lateral tibial tables (KN), greater trochanters (GT), anterior-superior iliac spines (IS), and gleno-humeral-joints (GH).

Pain during FES-assisted walking was registered by means of a 10-cm analog pain scale, while a value close to 10 means higher pain. The pain scale was presented to the subject three times (Stim1, Stim2, and Stim3).

### Experimental protocol

Before data collection, the subject had 5–7 s to reach steady motion on the treadmill. Data collections (3 min each) were performed five times during treadmill walking: before FES (Before), three times during FES (Stim1, Stim2, and Stim3) and after FES (After). The same protocol of data collection was used in the Control group where participants walked for 40 min on the treadmill without FES.

In the FES group, the experimenter increased the intensity of stimulation every 10 min (muscle by muscle, in the range 0–250 μs of impulse duration under the verbal control of the subject—every time up to the tolerant level of pain intensity). Each increase was performed during the first minute of each 3-min interval of data collection.

### Data analysis

The spatial coordinates of each marker were recorded, the body being represented as an interconnected chain of rigid segments. Kinematics data were filtered with a low-pass zero-phase shift Butterworth filter with a 5 Hz cut-off frequency. Stride length, walking frequency and velocity were estimated using the body mid-point (average of left and right GT, IS coordinates). This resulting point provides a good estimation of the center of mass (Courtine and Schieppati, 2003).

The elevation angle of each segment in the sagittal plane corresponds to the angle between the projected segment and the vertical and were computed as:

$$\begin{array}{l} \theta_{i\text{\_}sagittal}={\text{tan}}^{-1}\left[\left(X_{\text{id}}-X_{\text{ip}}\right)∕\left(Y_{\text{id}}-Y_{\text{ip}}\right)\right], \end{array}$$

where *X* and *Y* designate the coordinates of the proximal (p) and distal (d) markers for the *ith* frame of the acquisition. The elevation angle in the sagittal plane of the thigh (GT-KN), shank (KN-MAL) and foot (MAL-MTP) segments were calculated.

The elevation angle of the trunk in the sagittal, frontal, and horizontal planes corresponds to:

$$\begin{array}{l} \theta_{trank\text{\_}sagittal}={\text{tan}}^{-1}\left\{\frac{\left(\frac{\left(X_{\text{ IS\_left}}+X_{\text{ IS\_right}}\right)}{2}-\frac{\left(X_{\text{ GH\_left}}+X_{\text{ GH\_right}}\right)}{2}\right)}{\left(\frac{\left(Y_{\text{ IS\_left}}+Y_{\text{ IS\_right}}\right)}{2}-\frac{\left(Y_{\text{ GH\_left}}+Y_{\text{ GH\_right}}\right)}{2}\right)}\right\} \end{array}$$

and was calculated for each frame of the acquisition (Laroche et al., 2007). The trunk elevation angle in the frontal plane was computed with the same equation in the ZY plane and in the horizontal—in the XZ plane. Each trial was separated into gait cycles using the elevation angle of the lower limb axis (the line joining the MAL and GT), as described in Borghese et al. (1996). Stance phases were computed using the limb axis as described in Ivanenko et al. (2002) and was expressed in percentage of the gait cycle.

The coefficient of variation (CV) was calculated for each elevation angle to represent data variability and was calculated as the standard deviation divided by mean values across all steps of each subject during each 3 min of data collections (Bacarin et al., 2009).

### Statistics

Mean and Descriptive statistics included means and the SE of the mean. Paired *t*-test and ANOVA were used when appropriate to compare means. In particular, to evaluate the effects of FES on the amplitude of trunk oscillations and spatio-temporal parameters, the two-way ANOVA with first factor “FES” (Before, Stim1, Stim2, Stim3 and After) and the second factor “group”(FES, Control) was used. When significant effects were found, *post-hoc* Tukey's testing was conducted to identify the loci of these effects. The level of statistical significance was set at 0.05.

## Results

### General gait parameters

**Figure 1:**
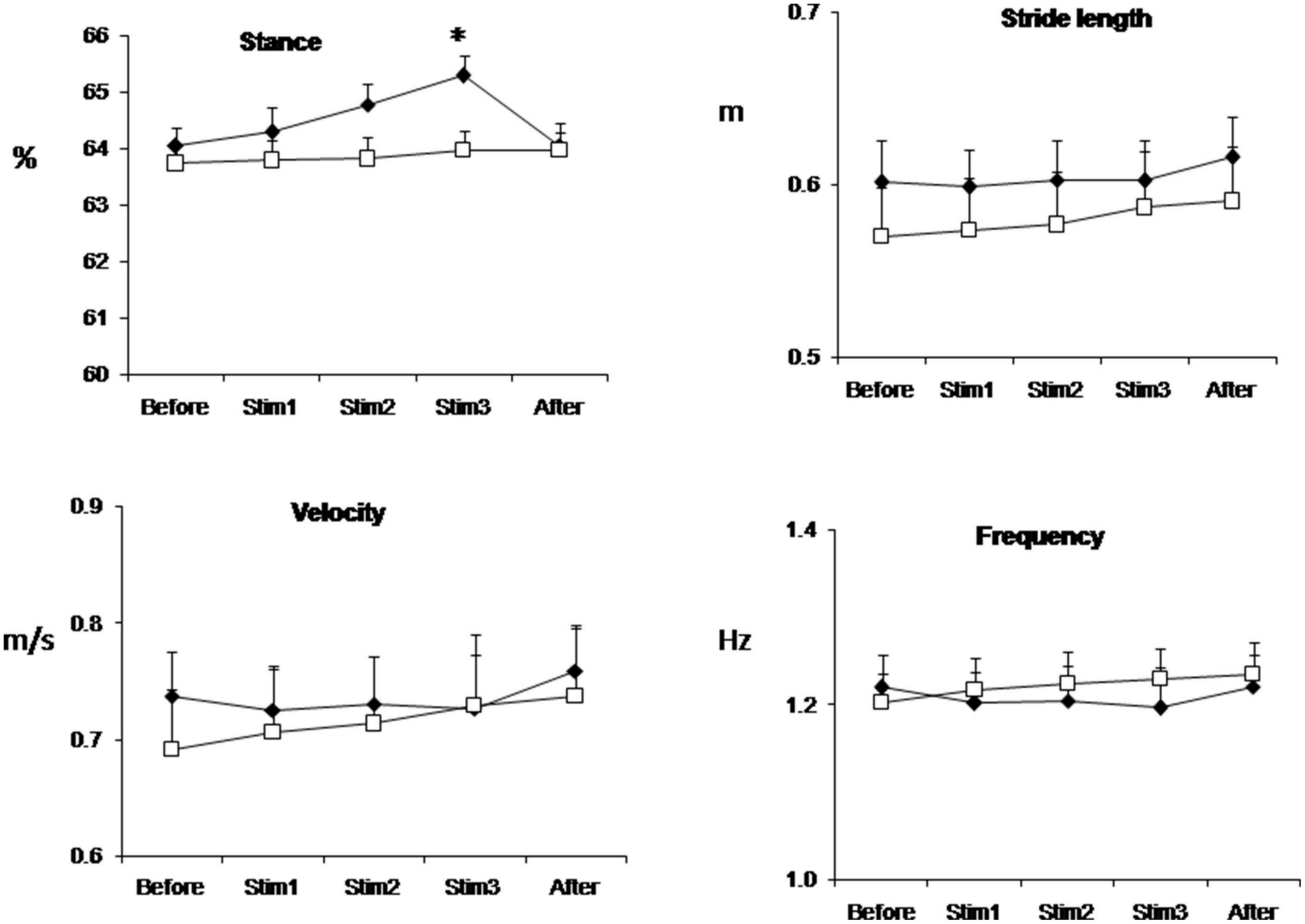
**Stance duration, stride length, velocity, and frequency of gait averaged across all trials and subjects in FES (black diamond) and Control (white square) groups**. Values are mean ± SE. ^*^p < 0.05.

Figure 1 shows the mean values (over all trials and subjects) of relative stance duration, stride length, walking velocity and step frequency in the Control and FES groups. FES significantly affects stance time: in the FES group, the relative duration of a stance during Stim3 conditions was at average 65.29 ± 0.36% of the cycle; that is a larger value than that of any of the Control group. Note, that stride length was about the same in the FES and Control groups for these conditions (0.60 ± 0.02 m and 0.59 ± 0.03 m in the FES and Control groups, correspondingly). Statistical analyses revealed significant interaction between groups and conditions for relative duration of the stance [ANOVA, *F*_(4, 28)_ = 6.21, *p* = 0.001]. *Post-hoc* tests showed that the relative duration of the stance in the FES group was significantly larger in the Stim3 than in the Before condition. Figure 1 also shows that control subjects exhibited the tendency of the well-known monotonic relationship of stride length and step frequency with speed increase (see Bernstein, 1935; Grillner, 1981; Winter and Scott, 1991).

### Trunk oscillations

**Figure 2:**
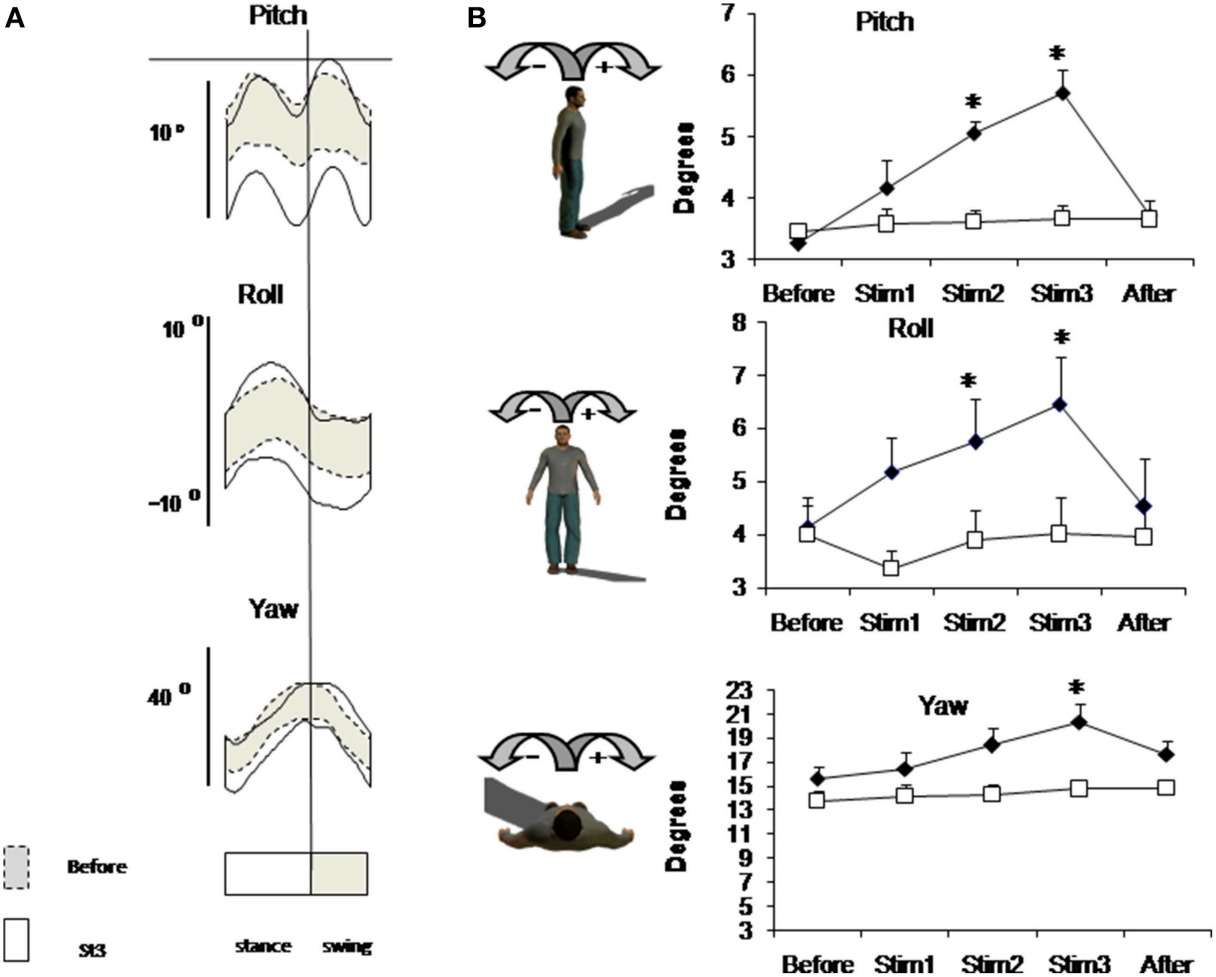
**Trunk oscillation evoked by FES**. **(A)** Trunk motion (±SD value) in one representative subject from FES group averaged across 3-min intervals of Before (dashed lines) and Stim3 (solid lines) periods. **(B)** Peak-to-peak amplitudes of trunk oscillations averaged across FES (black diamond) and across Control (white square) groups (mean ± SE). Asterisks indicate statistical difference in Stim condition in comparison with Before condition in FES group, ^*^p < 0.05.

Figure 2 shows mean individual (Figure 2A) and group mean data (Figure 2B) of trunk oscillations in sagittal (Pitch), frontal (Roll), and horizontal (Yaw) planes. Group mean data shows that before stimulation trunk oscillations were not different in Control and FES group (mean trunk oscillation in Before condition was in saggital plane 3.26 ± 0.14° and 3.45 ± 0.15°, in frontal plane 4.0 ± 0.55°, and 4.15 ± 0.55° and in horizontal plane 15.60 ± 0.57° and 13.78 ± 0.85° for FES and Control, respectively). Electrical stimulation of leg muscle significantly affects the amplitude of trunk oscillation: these amplitudes progressively increase in the FES group [ANOVA *F*_(4, 56)_ = 12.28, *p* = 0.0002; *F* = 6.48, *p* = 0.0002; *F* = 5.98, *p* = 0.0004 for Pitch, Roll and Yaw, correspondingly].

### Limb elevation angels in sagittal plane

**Figure 3:**
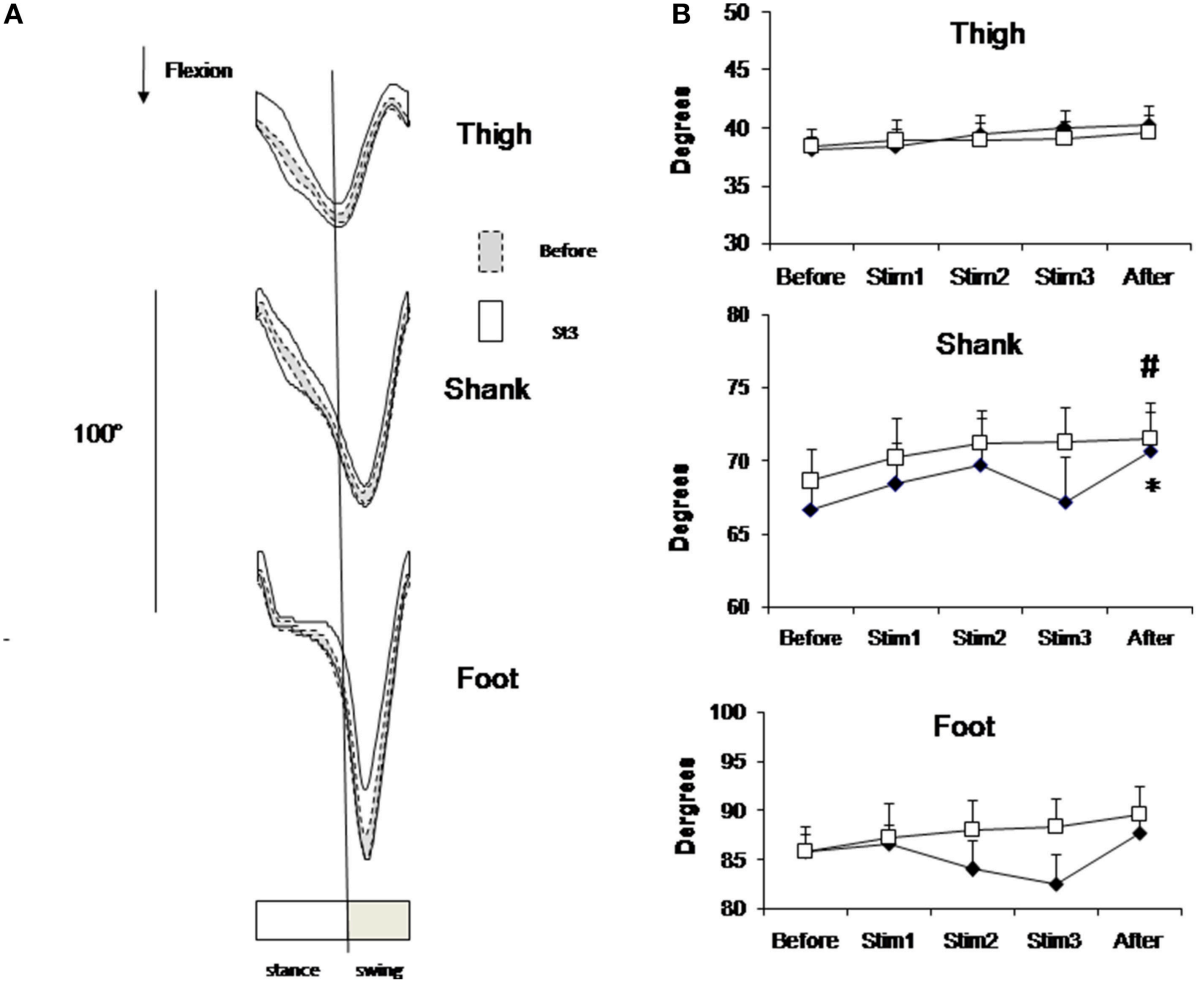
**Elevation angels in sagittal plane**. **(A)** Segment elevation angels (±SD value) in one representative subject from FES group averaged across 3-min intervals of Before (dashed lines) Stim3 (solid lines) periods. **(B)** Peak-to-peak amplitudes of elevation angels averaged across FES (black diamond) and Control (white square) groups (mean ± SE) Asterisk (^*^) indicates statistical difference in the condition in comparison with Before condition in FES group; sharp (^#^) indicates statistical difference—in the condition in comparison with Before condition in Control group (*p* < 0.05).

Figure 3 shows the profile of mean individual data (Figure 3A) and group mean data (Figure 3B) of limb elevation angels in sagittal plane. Due to stimulation, the profile of limb elevation angels didn't change, but the mean individual amplitude of foot angels decreased. Mean group data presented on Figure 3B also shows this tendency for distal joints, but these changes didn't approach the level of significance due to high variability of limb elevation angels due to FES. The CV of foot angle increased from 0.03 at Before up to 0.07 at Stim2 and 0.08 at Stim3 conditions (*t*-test, *p* < 0.01).

### Perceptual effects

All subjects in FES group reported the instability increase due to FES and the fatigue sensation afterwards. There were subjective reports during FES such as: “The locomotion is not free, my legs are out of my control, I feel my ankle joint blocked, “freezing,” muscles are fatigued, I have sensation as walking in flippers.” After the end of FES, several subjects sensed that the treadmill decelerated, and locomotion seemed “unusually light.” Actually, some participants approached the forward part of the treadmill belt during FES. Subjective pain rating increased up to 8 cm in the Stim 3 condition (Table 1).

**Table 1:** **Stimulation intensity (μs) of four muscles and the severity of pain for one typical subject in Stim1, Stim2 and Stim3 conditions**.

**Pain (cm)**	**Intensity of stimulation (duration of stimulation train, μs)**			
	**TA**	**GM**	**RF**	**B**
5.5	60.6	82.9	92.4	92.4
7.5	143.4	162.6	162.6	130.9
8.5	226.3	239.1	258.2	334.7

### After-effect

At the end of 40 min locomotion limb elevation angels appeared slightly increased in both groups in comparison with Before (Figure 3B). This increase approached the level of significance for shank angle in both groups (*t*-test, *p* < 0.05).

## Discussion

In this paper, we analyzed the effects of FES on the kinematics of a healthy subjects' locomotion. The most remarkable changes caused by FES are the increase of body oscillation, accompanied by an increase in stance time duration.

### Postural instability

Although the mechanical reason of increased trunk pitch could be the excessive contraction of quadriceps muscle, the increased trunk oscillation both in sagittal, frontal and horizontal planes indicates the decrease of locomotion stability and movement disturbance caused by FES. The disruption of the normal gait by FES manifests itself as an increase in stance time, providing an increased support time—another index of instability (Bernstein, 1935; Kirtley, 2006). The fact that these parameters returned to the norm immediately after FES are in line with this explanation. Pain, especially due to strong stimulation of the tibialis muscle could also distort the sensory feedback from muscle and skin receptors and there by the descending control from the CNS.

Comparing FES-assisted and non-assisted walking of one incomplete spinal injuries patient Ladouceur and Barbeau (2000) have also found FES-induced decrease of ankle plantar flexion by 5.6°, which is similar to our data of FES-assisted walking (Figure 3A). The tendency of ankle and shank joints to flex less in the swing phase during intensive FES is similar to the effect of under-flexion of the knee during fatigued walking (Bernstein, 1935). However, these effects could be of a different origin: in our study the TA muscle being stimulated along the swing phase is more pain sensitive (Table 1). To avoid pain, the subject could aim to dorsiflex the TA muscle less than would be seen in a natural gait pattern so as to decrease the TA stimulation time and thus the amount of pain. This, in turn, resulted in excessive dorsiflexion of the ankle, which leads to a decrease of push of force.

### Clinical application

FES is a commonly used clinical tool to improve walking ability due to its simplicity, low-cost, and strong muscle response, however, muscle fatigue is a major limiting factor in FES applications (Kralj et al., 1988; Karu et al., 1995). FES is shown to have a more significant effect in comparison with physiotherapy in walking speed increase during and after FES-assisted locomotor training (see review by Taylor et al., 2013). This notion is supported by the study of Khaslavskaia et al. (2002), which has shown that the changes in healthy participants in the TA MEP during locomotion were seen over 20 min following the cessation of the stimulation of the common peroneal nerve. It is now widely accepted, that FES improves ankle dorsiflexion in the long-term perspective, increasing the corticospinal excitability and that FES-assisted training “facilitates motor relearning” in patients (Ladouceur and Barbeau, 2000; Alon and Ring, 2003; Lindquist et al., 2007; Barrett et al., 2009; Daly et al., 2011). In the present study, intensive phasic electrical stimulation was applied to both ankles and hip antagonist muscles, and similarly to leg orthosis in neurological subjects. Similarly to clinical settings, the timing of stimulation was on-line controlled trough the feedback from the current knee joint angle in each stride (this way, in clinical practice, the pathological walking is aimed to be adjusted to the typical normal walking, then the “re-education” of pathological walking is expected). Our results indicate that, in healthy subjects, the postural component of locomotor activity was changed due to strong electrical stimulation of leg muscles, while the rhythmic component remains intact. It could be speculated, that the artificial nature of muscle contractions during FES-assisted walking in healthy subjects transfers the locomotor activity from involuntary to a more voluntary controlled movement. It could be concluded, that the functional role of FES for patients is the “adaption training” rather than “re-education” of pathological walking into the typical “normal” walking.

### Conflict of interest statement

The authors declare that the research was conducted in the absence of any commercial or financial relationships that could be construed as a potential conflict of interest.
